# Editorial: CCR5: A receptor at the center stage in infection

**DOI:** 10.3389/fimmu.2022.1054430

**Published:** 2022-10-18

**Authors:** Joel Henrique Ellwanger, Massimiliano Secchi, Julio Aliberti, Luca Vangelista

**Affiliations:** ^1^ Laboratory of Immunobiology and Immunogenetics, Department of Genetics, Universidade Federal do Rio Grande do Sul (UFRGS), Porto Alegre, Brazil; ^2^ Institute of Molecular Genetics, National Research Council, Pavia, Italy; ^3^ Office of AIDS Research, National Institutes of Health (NIH OAR), Bethesda, MD, United States; ^4^ Department of Biomedical Sciences, School of Medicine, Nazarbayev University, Astana, Kazakhstan

**Keywords:** CCR5, CCR5Δ32, gene-editing, COVID-19, HIV therapy, infection, maraviroc, leronlimab

## CCR5: As receptor that has shaped science

**Figure 1:**
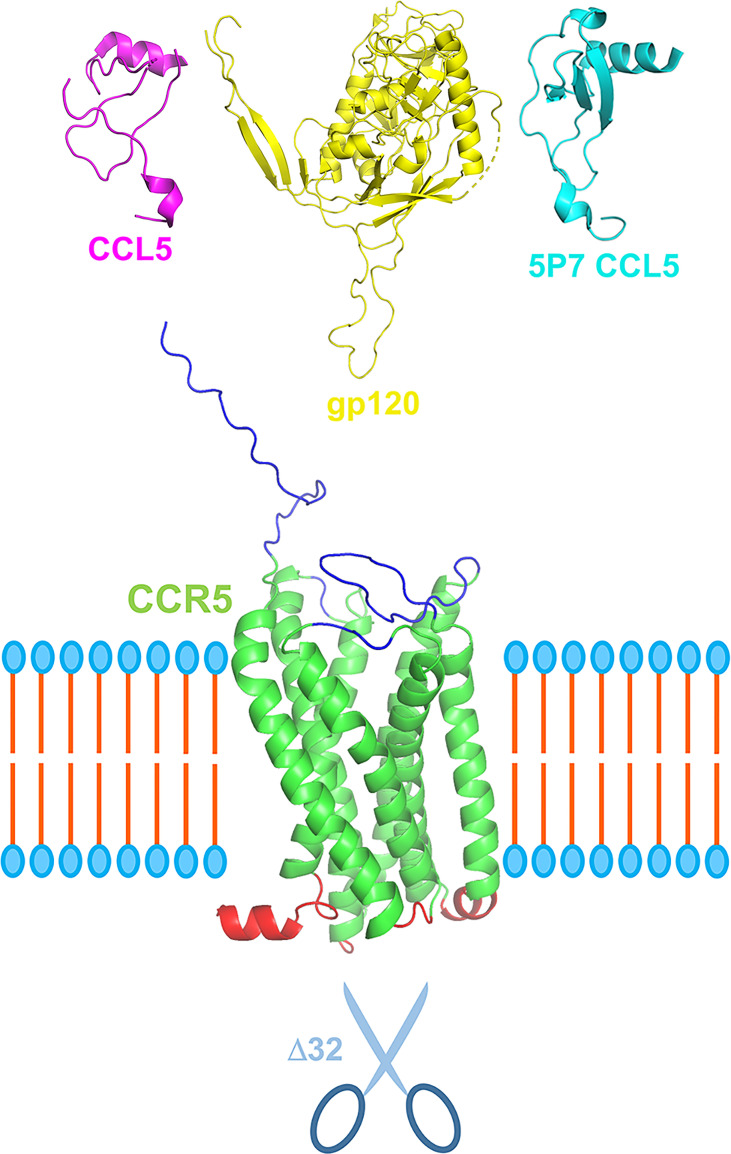
Schematic representation of CCR5 in its central role in pathophysiology. CCR5 (green), depicted here with extracellular portions in blue and intracellular in red, can be engaged by its natural ligands, e.g., CCL5 (purple), but also by HIV-1 gp120 (yellow) *via* its V3 loop. CCR5 antagonists, e.g., 5P7 CCL5 (light blue), can block the receptor in its inactive form preventing receptor internalization and engagement by HIV-1 gp120. More in general, CCR5 antagonists such as chemokine derivatives, small chemical compound (e.g., maraviroc) and monoclonal antibodies (e.g., leronlimab) could provide a therapeutic landscape for the array of infectious and inflammatory diseases in which CCR5 plays a central role. Scissors represent the CCR5 Δ32 deletion variant that confers resistance to HIV-1 infection and proved relevant for other CCR5-centered diseases. Three dimensional ribbon representation were generated using PyMOL: CCR5 and gp120 from PDB entry 6MEO, CCL5 from 7F1R, and 5P7 CCL5 from 5UIW. The relative sizes of the different proteins are not to scale. The cell membrane bilayer is schematized.

The human C-C chemokine receptor type 5 (CCR5) is mostly expressed on the surface of leukocytes, playing a pivotal role in inflammatory responses and other immune functions (**Figure 1**) (1, 2). In 1996, CCR5 was reported as the HIV-1 co-receptor (3), and the 32-nucleotide deletion in the CCR5 gene (CCR5Δ32) was reported as a resistance factor to HIV-1 infection (4, 5). These discoveries massively advanced HIV-1 research, bringing insights into resistance mechanisms against HIV-1 and leading to the development of new anti-viral therapies. The clinical use of the CCR5 antagonist maraviroc for HIV-1 treatment was approved in 2007, and cases of sustained remission of HIV-1 infection following stem-cell transplantation using CCR5Δ32 homozygous cells were reported in the following years (e.g., the Berlin Patient in 2009 and the London Patient in 2019) (2, 6, 7).

CCR5 was initially studied in different populations in the context of HIV-1 infection. More recently, it has become clear that CCR5 influences various health and pathological conditions, including infectious diseases other than HIV-1 infection. For example, CCR5 and its agonists participate in the immune responses to Zika virus (8), severe acute respiratory syndrome coronavirus 2 (SARS-CoV-2) (9), *Schistosoma* spp. (10), among other pathogens (2, 10), and the CCR5 Δ32 variant is a critical risk factor for symptomatic West Nile virus infection (11, 12). Moreover, human pathogens, such as *Toxoplasma gondii* produce CCR5-binding molecules that can affect immune response to infection as well as block R5 tropic HIV-1 entry in CCR5-expressing cells (13–15).

Therapies involving CCR5 blockade have advanced substantially, with the potential to be applied in infectious and non-infectious diseases (1, 16, 17). CCR5 also drives several debates, including those on gene-editing technologies (18) and chemokine system redundancy and robustness (19). CCR5 is exemplary of how a single molecule may shape different research fields, and many advances related to CCR5 continue to be made, as highlighted by articles published on this Research Topic.

## New contributions to CCR5 research

This Research Topic brings together important contributions to the understanding of CCR5 biology and participation of this receptor in numerous aspects of infectious diseases. Using machine learning methods and data from human samples, Patterson et al. explored the immune spectrum of SARS-CoV-2 infection, including the impact of CCR5 and its ligands on COVID-19. Analyzing different patient profiles, the study showed that severe COVID-19 cases are characterized by excessive inflammation and dysregulated T cell activity. Patterson et al. also characterized the immune profile of post-acute sequelae of COVID-19 (PASC) patients. In brief, this study reports important data to the understanding of the participation of CCR5 and other immune molecules in the COVID-19 spectrum, including the intriguing PASC cases, and describes tolls to predict COVID-19-related immune outcomes.

Exploring basic aspects of CCR5-HIV-1 interactions, the article by Picton et al. evidenced the genetic predisposition to lower CCR5 expression in individuals who naturally control HIV-1, based on data from black South African individuals. This is a relevant and updated contribution to the understanding of differential progression of HIV-1 infection, especially by focusing on a sub-Saharan population. The debate about the CCR5-HIV-1 interactions was also advanced and updated by Jasinska et al., who addressed the CCR5 as a co-receptor for HIV-1 and simian immunodeficiency viruses in an interesting review. This is a great reference for those seeking accurate and relevant information concerning CCR5 in evolutionary and host-pathogen interaction perspectives. In a complementary way, Mohamed et al. reviewed the efficacy of CCR5-based HIV-1 therapies, also describing important information concerning CCR5 biology. Several mechanisms to control HIV-1 infection progression are discussed in the article, including the use of small-molecule inhibitors, anti-CCR5 antibodies, disruption of CCR5 expression, and *CCR5*-editing strategies.

Considering the next generation of HIV-1 therapies, the work by Karuppusamy et al. described important data on the use of *CCR5*-edited CD34^+^CD90^+^ hematopoietic stem cells as a graft source for HIV-1 gene therapy. This is an exciting and detailed study with numerous *in vitro* and *in vivo* (animal) experiments. The potentialities of *CCR5*-editing were also evaluated by Scheller et al., who showed in a proof-of-concept study that targeting cells using CRISPR-Cas9 mediated HDR (homology directed repair) enables the selection of mutant cells that are CCR5 deficient and highly resistant to HIV-1 infection. Together, results from Karuppusamy et al. and Scheller et al. move forward the research on HIV-1 gene therapies. Amerzhanova and Vangelista explored structural details of the occupancy of CCR5 orthosteric site by several antagonist ligands, focusing on the 3D modeling analysis of CCL5 mutants, and discussing their likely contributions to HIV-1 therapy as well as the entire spectrum of diseases where this receptor is central. Classic and novel strategies to block G protein-coupled receptors (GPCRs) have been discussed and related to CCR5 blockade. Finally, the study by Chang et al. also brings an important contribution to HIV-1 treatment investigation based on CCR5 blockade. With data obtained from humans and non-human primates, the authors evidenced increased peripheral blood CCR5^+^CD4^+^ T cells following treatment with leronlimab, a promising anti-CCR5 antibody. Chang et al. also bring contributions concerning the monitoring of the use of anti-CCR5 therapeutic antibodies and the impacts of CCR5 blockade on the immune function.

Kulmann-Leal et al. reviewed the impacts of CCR5Δ32 on the Brazilian population, discussing how the colonization of Brazil shaped the CCR5Δ32 distribution in different regions of the country. The article showed that CCR5Δ32 affects cancer, inflammatory conditions, and infectious diseases heterogeneously in Brazilians, with particular influences on each disease. The influences of CCR5 on influenza virus infection were reviewed by Ferrero et al.; with an informative and didactic figure, the authors addressed specifically the dual role of CCR5 during influenza-related immune responses, bringing insights into treatment opportunities. Taken together, these two reviews show how the CCR5 impact on different diseases is relatively complex and cannot be generalized. In this context, Bauss et al. used the CCR5 as a study model to combat biological reductionism. Using a set of bioinformatics tools and student participation, the article evidenced the biological complexity of CCR5, highlighting its involvement in numerous biological contexts, beyond HIV-1 infection. This article brings contributions to a deep understanding of CCR5 functions in the human body, and demonstrates how CCR5 can be used as a tool for addressing biological and social debates.

## Conclusion

This Research Topic highlights studies addressing different aspects of CCR5 in infection, contributing to the understanding of CCR5 participation in immune responses (in health and disease contexts), and reporting updated information and new data on therapeutic potentials of CCR5 modulation and gene-editing. This Research Topic will be useful to readers from multiple fields, and confirm that CCR5 continues to be under the spotlight of research involving immunology and infectious diseases.

## Author contributions

JE wrote the first draft of the manuscript. LV prepared the figure. LV, MS and JA edited and complemented the text. LV supervised the work. All authors revised and approved the manuscript.

## Funding

JE receives a postdoctoral fellowship from Coordenação de Aperfeiçoamento de Pessoal de Nível Superior (Programa Nacional de Pós-Doutorado – PNPD/CAPES, Brazil). LV acknowledges support from Nazarbayev University grant 021220FD2551 “Expanding the therapeutic landscape of CCR5 blockade and CCL5 engineering”.

## Acknowledgments

The editors are grateful to all authors who participated in this Research Topic and to reviewers who contributed substantially to improving the quality of the articles with critical comments and relevant suggestions.

## Conflict of interest

The authors declare that the research was conducted in the absence of any commercial or financial relationships that could be construed as a potential conflict of interest.

## Publisher’s note

All claims expressed in this article are solely those of the authors and do not necessarily represent those of their affiliated organizations, or those of the publisher, the editors and the reviewers. Any product that may be evaluated in this article, or claim that may be made by its manufacturer, is not guaranteed or endorsed by the publisher.

## References

[1] Martin-Blondel G Brassat D Bauer J Lassmann H Liblau RS. CCR5 blockade for neuroinflammatory diseases–beyond control of HIV. Nat Rev Neurol (2016) 12(2):95–105. doi: 10.1038/nrneurol.2015.248 26782333

[2] Ellwanger JH Kulmann-Leal B Kaminski VL Rodrigues AG Bragatte MAS Chies JAB. Beyond HIV infection: Neglected and varied impacts of CCR5 and CCR5Δ32 on viral diseases. Virus Res (2020) 286:198040. doi: 10.1016/j.virusres.2020.198040 32479976 PMC7260533

[3] Dragic T Litwin V Allaway GP Martin SR Huang Y Nagashima KA. HIV-1 entry into CD4^+^ cells is mediated by the chemokine receptor CC-CKR-5. Nature (1996) 381(6584):667–73. doi: 10.1038/381667a0 8649512

[4] Huang Y Paxton WA Wolinsky SM Neumann AU Zhang L He T. The role of a mutant CCR5 allele in HIV-1 transmission and disease progression. Nat Med (1996) 2(11):1240–3. doi: 10.1038/nm1196-1240 8898752

[5] Samson M Libert F Doranz BJ Rucker J Liesnard C Farber CM. Resistance to HIV-1 infection in caucasian individuals bearing mutant alleles of the CCR-5 chemokine receptor gene. Nature (1996) 382(6593):722–5. doi: 10.1038/382722a0 8751444

[6] Hütter G Nowak D Mossner M Ganepola S Müssig A Allers K. Long-term control of HIV by *CCR5* Delta32/Delta32 stem-cell transplantation. N Engl J Med (2009) 360(7):692–8. doi: 10.1056/NEJMoa0802905 19213682

[7] Gupta RK Abdul-Jawad S McCoy LE Mok HP Peppa D Salgado M. HIV-1 remission following CCR5Δ32/Δ32 haematopoietic stem-cell transplantation. Nature (2019) 568(7751):244–8. doi: 10.1038/s41586-019-1027-4 PMC7275870 30836379

[8] Mladinich MC Conde JN Schutt WR Sohn SY Mackow ER. Blockade of autocrine CCL5 responses inhibits zika virus persistence and spread in human brain microvascular endothelial cells. mBio (2021) 12(4):e0196221. doi: 10.1128/mBio.01962-21 34399621 PMC8406327

[9] Chua RL Lukassen S Trump S Hennig BP Wendisch D Pott F. COVID-19 severity correlates with airway epithelium-immune cell interactions identified by single-cell analysis. Nat Biotechnol (2020) 38(8):970–9. doi: 10.1038/s41587-020-0602-4 32591762

[10] Ellwanger JH Kaminski VL Rodrigues AG Kulmann-Leal B Chies JAB. CCR5 and CCR5Δ32 in bacterial and parasitic infections: Thinking chemokine receptors outside the HIV box. Int J Immunogenet (2020) 47(3):261–85. doi: 10.1111/iji.12485 32212259

[11] Glass WG McDermott DH Lim JK Lekhong S Yu SF Frank WA. CCR5 deficiency increases risk of symptomatic West Nile virus infection. J Exp Med (2006) 203(1):35–40. doi: 10.1084/jem.20051970 16418398 PMC2118086

[12] Lim JK Louie CY Glaser C Jean C Johnson B Johnson H. Genetic deficiency of chemokine receptor CCR5 is a strong risk factor for symptomatic West Nile virus infection: a meta-analysis of 4 cohorts in the US epidemic. J Infect Dis (2008) 197(2):262–5. doi: 10.1086/524691 18179388

[13] Aliberti J Valenzuela JG Carruthers VB Hieny S Andersen J Charest H. Molecular mimicry of a CCR5 binding-domain in the microbial activation of dendritic cells. Nat Immunol (2003) 4(5):485–90. doi: 10.1038/ni915 12665855

[14] Golding H Aliberti J King LR Manischewitz J Andersen J Valenzuela J. Inhibition of HIV-1 infection by a CCR5-binding cyclophilin from toxoplasma gondii. Blood (2003) 102(9):3280–6. doi: 10.1182/blood-2003-04-1096 12855560

[15] Moreno-Fernandez ME Aliberti J Groeneweg S Köhl J Chougnet CA. A novel role for the receptor of the complement cleavage fragment C5a, C5aR1, in CCR5-mediated entry of HIV into macrophages. AIDS Res Hum Retroviruses (2016) 32(4):399–408. doi: 10.1089/AID.2015.0099 26537334 PMC4817595

[16] Vangelista L Vento S. The expanding therapeutic perspective of CCR5 blockade. Front Immunol (2018) 8:1981. doi: 10.3389/fimmu.2017.01981 29375583 PMC5770570

[17] Poddighe D Vangelista L. *Staphylococcus aureus* infection and persistence in chronic rhinosinusitis: Focus on leukocidin ED. Toxins (Basel) (2020) 12(11):678. doi: 10.3390/toxins12110678 PMC7692112 33126405

[18] Rosenbaum L. The future of gene editing - toward scientific and social consensus. N Engl J Med (2019) 380(10):971–5. doi: 10.1056/NEJMms1817082 30649992

[19] Ellwanger JH Kaminski VL Chies JAB. What we say and what we mean when we say redundancy and robustness of the chemokine system - how CCR5 challenges these concepts. Immunol Cell Biol (2020) 98(1):22–7. doi: 10.1111/imcb.12291 31613403

